# Consumption of Psychiatric Drugs in Primary Care during the COVID-19 Pandemic

**DOI:** 10.3390/ijerph19084782

**Published:** 2022-04-14

**Authors:** María del Carmen González-López, Virginia Díaz-Calvo, Carlos Ruíz-González, Bruno José Nievas-Soriano, Belén Rebollo-Lavado, Tesifón Parrón-Carreño

**Affiliations:** 1 Primary Health Care District of Almeria, Andalusian Health Service, 04006 Almeria, Spain; mariac.gonzalez.lopez.sspa@juntadeandalucia.es (M.d.C.G.-L.); vdiazca@gmail.com (V.D.-C.); carlos231993@gmail.com (C.R.-G.); 2 Department of Nursing Science, Physiotherapy and Medicine, University of Almería, 04006 Almeria, Spain; tpc468@ual.es; 3 Neurology Department, Badajoz Universitary Hospital, 06080 Badajoz, Spain; belen.rebollo.93@gmail.com

**Keywords:** psychiatric drugs, primary health care, COVID-19, drug consumption, drug prescription, antidepressants, anxiolytics, hypnotic-sedatives

## Abstract

Background: The main objective of this research was to analyze whether there were changes in the use of antidepressants, anxiolytics, and hypnotic-sedative drugs, in the context of primary health care, during the COVID-19 pandemic compared to the pre-pandemic period. We further sought to study consumption in vulnerable population groups. Methods: A cross-sectional observational study was performed in a primary health district of Spain. The data were obtained from the Andalusian Public Health System database, for the pre-COVID-19 period, from March 2019 to February 2020, and for the COVID-19 period, from March 2020 to February 2021. Univariant and bivariant analyses were performed. The effect size was measured using the Rosenthal test. Results: While the total number of medical prescriptions decreased by 2.5% in the COVID-19 period, the prescriptions of psychiatric drugs increased by 6.1%. The increase in the dose consumption per 1000 inhabitants (DHD) was highest for anxiolytics (7.2%), followed by hypnotic-sedatives (5.6%) and antidepressants (3.7%). The consumption of antidepressants, anxiolytics, and sedative-hypnotic drugs was higher in women, older people, and rural areas and lower in areas with social transformation needs, with these differences being statistically significant. Conclusions: The consumption of psychiatric drugs has increased over the COVID-19 pandemic, especially in women, older people, and rural areas. Thus, we should reflect on the adequate use of these drugs.

## 1. Introduction

Mental disorders affect up to 25% of the population, representing one of the main challenges of public health [1,2]. Major depressive disorders are one of the main causes of disability, diminishing the quality of life and increasing the burden of care in the health care systems and the risk of premature mortality [3,4,5]. These disorders could affect more than 300 million persons in the world [6,7,8,9]. The most prevalent of these disorders is depression. Its prevalence in the European region is 6.4% [10], but there are considerable geographic variabilities, with figures between 5% and 10%, showing important differences among countries [6,9,10]. In Spain, the prevalence is 6.7%, being more frequent in women, in persons with permanent disabilities, and persons with low economic levels [11].

According to the World Health Organization (WHO), mental disorders have increased during the COVID-19 pandemic, reaching a prevalence of 33% among adults, due to problems such as anxiety or stress [12]. In Spain, the prevalence of mental disorders has also increased [13], and the Mental Health Survey of February 2021 showed that a total of 22% of the surveyed citizens had a depressive disorder [14]. According to a report by the Spanish Health Ministry, the mean annual increase in the use of antidepressants in Spain, over thirteen years, was 4.05 units consumed per 1000 inhabitants (DHD) [15].

Health care systems have become more burdened as mental distress and disorders have significantly increased since the beginning of the COVID-19 pandemic [10,11,12,13,14,16]. This has entailed a higher consumption of psychiatric drugs, such as antidepressants, or drugs for the treatment of anxiety and insomnia [17]. In the United Kingdom, the use of psychiatric drugs has increased since the beginning of the COVID-19 pandemic [18]. In Spain, this increase was more prominent in women and depressed areas [14,19,20].

Given the impact of these disorders on different areas, it is important to find out if there is an increased use of psychiatric drugs in the primary health care context. Furthermore, it is also essential to describe which specific subgroups of these drugs, such as antidepressants, anxiolytics, or hypnotic-sedative drugs, have increased the most. This information can be used to develop strategies and health policies that can help to manage the increase in mental health disorders in the adverse context of the COVID-19 pandemic.

Therefore, the main objective of this research was to analyze whether there were changes in the use of antidepressants, anxiolytics, and hypnotic-sedative drugs, in the context of primary health care, during the COVID-19 pandemic compared to the pre-pandemic period. We further sought to study the impact of the use of these psychiatric drugs in the most vulnerable population groups and to establish the influence of sociodemographic factors on the use of these drugs.

## 2. Materials and Methods

### 2.1. Study Population

A cross-sectional observational study was performed in the Primary Health District of Almeria, in the country of Spain, with a population of 304,182 inhabitants, distributed among 18 primary health care centers, 11 of which are located in urban areas and the other 7 in rural areas. A total of 3 of these primary health care centers were ubicated in areas with social transformation needs. As the number of prescriptions in persons under 25 years was low, this population was excluded from the research.

### 2.2. Data Collection

The data were obtained from the FARMA database (Andalusian Public Health System, Spain), which records the total number of prescriptions that have been filled and picked up by the patients in the pharmacies, within two periods: the pre-COVID-19 period, from March 2019 to February 2020, with a total of 4,883,055 prescriptions, 549,334 of them which were psychiatric drugs; and the COVID-19 period, from March 2020 to February 2021, with a total of 4,762,419 prescriptions, 582,736 of which were psychiatric drugs.

The drugs analyzed were classified according to the Anatomical Therapeutic Chemical Classification (ATC). The analyzed groups were N06A (antidepressants), N05B (anxiolytics), and N05C (hypnotic-sedatives). The ATC establishes a Defined Daily Dose (DDD) for each active ingredient [21], which describes the daily maintenance dose of a drug when used for its main indication, which is listed on their website [22]. The Defined Daily Dose (DDD) was established by the Collaborating Centre for Drug Statistics Methodology, under the World Health Organization, for the statistical study of drug consumption [21]. The DDD is defined as the maintenance dose per day for an adult when the drug is used for its main indication. The Defined Daily Dose per 1000 inhabitants per day (DHD) allows us to measure the use of different drugs and to make comparisons among different geographical areas and times [23,24,25]. In drug utilization studies, to measure the degree of exposure of the population to drugs, a consumption rate is used, Defined Daily Doses per number of inhabitants per day (DHD): number of DDD/1000 inhabitants/day. This parameter estimates the number of patients treated daily with a given drug [25].

### 2.3. Data Analysis

To perform data analysis, seven age strata were used for most of the analyses, although for some of them, only two age strata were used (under 65 and 65 and older), as in this last stratum, the use of drugs was considerably higher. Other analyzed variables were sex, urban or rural areas, and living in areas with social transformation needs (STN). These are urban areas in which many of the inhabitants experience conditions of social exclusion, severe poverty, and social marginalization.

For quantitative variables, central tendency and dispersion measures were used. For qualitative variables, absolute frequencies and percentages were used. For means and proportions, 95% confidence intervals (CI) were calculated. The goodness of fit to normality for the variables was calculated using the Kolmogorov–Smirnov test.

To perform bivariate analysis, the nonparametric Mann–Whitney test for unpaired data and the Wilcoxon test for paired data were used. Statistical analyses were performed using SPSS version 26 (IBM Inc., Armonk, NY, USA). The effect size was measured using the Rosenthal test, whose result was assessed according to Cohen’s classification. Thus, <0.3 was considered as a low effect, 0.3–0.5 as a moderate effect, and >0.5 as a high effect [26].

### 2.4. Ethical Aspects and Review Board Approval

Regarding ethical aspects and review board approval, this study used prescription records previously registered in a database. The data were collected and pooled, and no personal information was used, so data were anonymous, and no informed consent was required. All the collected data were processed according to Regulation (EU) No 679/2016 of the European Parliament; to General Registry for the Protection of Personal Data under the Spanish Data Protection Agency Law of 27 April 2016; and to Spanish Organic Law 3/2018, of 5 December, regulating protection of personal data and digital rights warranty. All the procedures described in this study were approved by the Ethics and Research Committee of the Primary Health Care of Almeria (Spain).

## 3. Results

The pre-COVID-19 period showed a total of 4,883,055 prescriptions, 549,334 of which were psychiatric drugs included in the three subgroups defined. The COVID-19 period showed a total of 4,762,419 prescriptions, 582,736 of which were psychiatric drugs. Thus, while the total number of medical prescriptions decreased by 2.5% between both periods, the prescription of psychiatric drugs increased by 6.1%. A total of 49% of the patients analyzed were women, and 67% lived in urban areas. A total of 20% of the patients analyzed lived in areas with social transformation needs.

**Figure 1:**
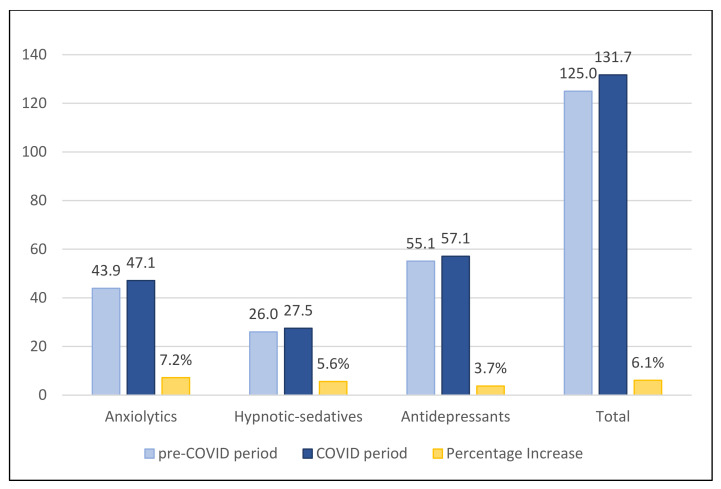
DHD of the psychiatric drugs in pre-COVID-19 and COVID-19 periods, and percentage increase.

Regarding the DHD (Figure 1), there was an increase in the three subgroups of psychiatric drugs in the COVID-19 period of 6.64 units of DHD. The biggest increase was found in the anxiolytics subgroup.

When analyzing the use of psychiatric drugs regarding sex and age (Table 1), the DHD was higher in women in the COVID-19 period for the three subgroups. The highest consumption of anxiolytics was found among women between 75 and 84 years, while the hypnotic-sedatives and the antidepressants were most frequently consumed among women older than 84 years.

Regarding the use of psychiatric drugs in persons older than 65 years (Table 2), the DHDs of the three subgroups of the psychiatric drugs were higher in the persons older than 65 years when compared with those under 65 years, in both periods, for men and women. These differences were statistically significant in all the groups, except for the anxiolytic subgroup, in men.

The total consumption of the three subgroups was significantly higher in the COVID-19 period (Table 3). The biggest difference was found in the anxiolytic subgroup. The effect size was highest for the anxiolytics subgroup, followed by the hypnotic-sedative subgroup. The antidepressants were in the moderate-effect-size area, while the other two subgroups were in the large-effect-size area, according to Cohen’s classification.

Regarding the use of psychiatric drugs in urban and rural areas (Table 4), the total consumption of anxiolytic and hypnotic-sedative drugs was significantly higher in rural areas during the COVID-19 period. The biggest difference in the means of the rural areas was found in the anxiolytics subgroup, with 3.8 points of difference between the means. The effect size was highest for anxiolytics, with 0.84 points in the Rosenthal test, followed by the hypnotic-sedatives subgroup. Both were in the large-effect area, while the effect size of the antidepressants was in the moderate area.

The total consumption in the three subgroups of psychiatric drugs was significantly higher in urban areas during the COVID-19 period. The biggest difference between the mean values was found in the anxiolytics subgroup, with 3.3 points, followed by the antidepressants. The effect size was highest for anxiolytics, with 0.77 points in the Rosenthal test, followed by the hypnotic-sedatives subgroup. The three subgroups were in the large-effect-size area, according to Cohen’s classification.

Regarding the use of psychiatric drugs in areas with social transformation needs (Table 5), the consumption of anxiolytics and hypnotic-sedatives was higher in the COVID-19 period, while the consumption of antidepressants was lower, albeit no statistically significant differences were found in this last case. The biggest difference between means was found in the anxiolytics subgroup. The effect size was highest for this same subgroup, with a value of 0.55, being in the large effect size area. The antidepressant subgroup was in the moderate-effect area, and the hypnotic-sedatives remained in the low-effect area.

In areas with no social transformation needs, the total consumption of anxiolytics, hypnotic-sedatives, and antidepressants was significantly higher in the COVID-19 period. The biggest difference between means was found in the anxiolytics subgroup, followed by the antidepressants subgroup. The effect size was highest for the anxiolytics, with 0.85 points in the Rosenthal test, followed by the hypnotic-sedatives. All of them were in the large-effect-size area.

The analysis of the consumption of the psychiatric drugs, regarding sex (Table 6), showed that the consumption of anxiolytics and hypnotic-sedatives was significantly higher in women during the COVID-19 period. The consumption of antidepressants was also higher, but the difference was not statistically significant. The biggest difference between means was found in the anxiolytics subgroup, followed by the hypnotic-sedatives subgroup. The effect size was highest for the anxiolytics subgroup, with 0.83 points in the Rosenthal test, followed by the hypnotic-sedatives subgroup. Thus, both subgroups were in the large-effect-size area, while the antidepressants were in the moderate area.

Regarding men, the consumption of the three subgroups was significantly higher during the COVID-19 period. The biggest difference between means was found in the anxiolytics subgroup, followed by the antidepressants. The effect size was highest for the hypnotic-sedatives subgroup, followed by the anxiolytics subgroup. The three subgroups were in the large-effect-size area, according to Cohen’s classification.

The analysis of the consumption, when comparing both sexes in both periods (Table 7), showed statistically significant differences between men and women during the COVID-19 period, for anxiolytics and antidepressants subgroups. In the pre-COVID-19 period, differences were found for the hypnotic-sedatives and the antidepressants subgroups, with consumption being higher in women in both periods. The effect sizes were in the moderate and low areas.

When comparing urban and rural areas in both periods (Table 7), the analyses showed that the consumption was higher in the rural areas, but no statistically significant differences were found for any period or area. The effect sizes were in the moderate and low areas.

When comparing areas with social transformation needs (STN) with areas with no needs (Table 7), the analyses showed statistically significant differences during the COVID-19 period for the antidepressants subgroups, with consumption being higher in the areas with no social transformation needs. The effect sizes were in the moderate and low areas.

## 4. Discussion

The main objective of this research was to analyze whether there were changes in the use of antidepressants, anxiolytics, and hypnotic-sedative drugs, in the context of primary health care, during the COVID-19 pandemic compared to the pre-pandemic period.

The results of our research show that, during the COVID-19 period, the consumption of the three subgroups of psychiatric drugs analyzed increased when compared to the pre-COVID-19 period. The increase in the use of these drugs in the COVID-19 period was 2.6 times higher than expected when compared to previous years [15]; thus, it seems logical to think that this increase could be related to the COVID-19 pandemic. In other research performed by the Spanish Health Ministry [27], the increase in the subgroup of anxiolytics was 2.6%, and the hypnotic-sedative subgroup saw an increase of 1.5%, both of which are lower than the values obtained in our findings. Nevertheless, the DHD consumption of these two subgroups of psychiatric drugs was lower in our research. Other research performed in some specific regions of Spain [20] showed that the consumption of anxiolytic drugs increased a total of 3.8% during the COVID-19 period. Again, the DHD consumption of our research was lower. A plausible explanation is that our research was focused on primary health care, so the hospital prescriptions were not analyzed, and the total figures could have been lower.

The Spanish Mental Health Survey during the COVID-19 pandemic [14] showed that, from the beginning of the pandemic, a total of 6.4% of the Spanish population has consulted a mental health professional. A total of 44% of these persons suffered from anxiety symptoms, 35% suffered from depression symptoms, and most of them were women. This research stated that a total of 5.8% of the Spanish population had received psychiatric treatments, especially anxiolytics and antidepressants. In the United States, other authors have described an increase of 21% in the use of psychiatric drugs [17], with this increase being 41% in antidepressants, 19% in hypnotic-sedatives, and 18% in anxiolytics. While similar findings have been described in more countries, such as the United Kingdom [18], in other countries, such as Portugal, the use of psychiatric drugs has decreased [28]. According to the authors, this may be a consequence of the lower number of medical consultations during the pandemic, due to the mobility restrictions. The findings of our research, therefore, agree with the increases described by these authors.

We further sought to establish the influence of some sociodemographic factors in the use of these drugs. Regarding sex, other research has described that the consumption in the COVID-19 period was higher in the three subgroups of psychiatric drugs, with a DHD of 64.5 for anxiolytics (83.5 in women and 43.4 in men); a DHD of 40.2 for hypnotic-sedatives (52.2 in women and 26.9 in men); and a DHD of 98.8 in antidepressants, where the consumption was almost three times higher in women [29]. In this last case, we must consider that, during the COVID-19 period, more than half of the mental health medical consultations were for women [14]. The consumption described in this previous research was higher than our findings, as that research was focused on persons older than 40 years. In our research, the consumption of the three subgroups of psychiatric drugs was higher in women during the COVID-19 period.

Regarding age, in our research we found that the consumption of psychiatric drugs increased with age, being especially high in persons older than 65 years in both sexes. In the anxiolytics subgroup, in our research, the highest consumption was found among women of 75–84 years. In the hypnotic-sedative subgroup, the highest consumption was found in women older than 85 years, who used these drugs more than twice as often as those in the 75–84 strata. These figures agree with the findings of other research performed in Spain, where the authors describe that anxiolytics consumption increased with age, especially in women between 60 and 89 years [29]. In this research, the consumption of hypnotic-sedatives in persons older than 40 years was 40.2 DHD (52.2 in women and 26.9 in men), increasing with age and being more common in women, until the 80–84-year strata. The persons older than 40 years showed a DHD of 98.9 for antidepressants, with this figure being three times higher in women between 70 and 74 years when compared to men. The biggest consumption figures were found in women between 80 and 84 years [29].

Regarding the consumption in urban and rural areas, our research showed that it was higher in the latter areas for the three subgroups of psychiatric drugs. This has been also described in other research, which stated that psychiatric drugs are more frequently consumed in rural areas, independent of age [29].

We further sought to study the impact of the use of these psychiatric drugs on the most vulnerable population groups. One interesting finding was that in the areas with social transformation needs, the consumption of the three subgroups of psychiatric drugs was lower, with this difference being statistically significant for the antidepressants. This finding does not match that of other research [18,28], which states that the consumption of these drugs is higher in populations with lower socioeconomic levels. For example, in Spain, it is estimated that a total of 3.6% of persons with a high socioeconomic level consume psychiatric drugs, while 9.8% of low-socioeconomic-level persons consume them. Another example is that, since the beginning of the COVID-19 pandemic, a total of 1% of persons of a high socioeconomic level have received treatment for depression, while this treatment has been prescribed for 3.9% of persons of low socioeconomic level [14]. According to other research, there is a social gradient associated with the consumption of antidepressants and hypnotic-sedatives as consumption usually increases as economic level decreases [29]. A possible explanation for our findings is that, in the referred research, the authors refer to low-socioeconomic populations, and we analyzed areas with social transformation needs. These areas comprise immigrants, many of which reside in them illegally, so many of them do not attend primary health care centers, and medical prescriptions are required to obtain psychiatric drugs. This could explain the lower consumption figures found in these areas.

Our research had some limitations that must be considered. One of them is that our study was performed using the database of the Andalusian Public Health Service for primary health care. Therefore, the prescriptions performed in private practice or the hospital of reference of the area were not included. Therefore, the real increase in the prescriptions could have been larger. It would have been useful to find out the real consumption of the drugs analyzed in this research. We cannot assume that all the prescribed drugs were consumed by the patients, but we understand that the potential difference between the prescription and the real consumption, in both periods, must be similar, as the population was the same. The antipsychotic drugs were not included in this research, as these drugs constitute a therapeutic group aimed at more severe pathologies, with a longer-lasting evolution. Thus, we understood that the analysis of these drugs in a short period would not be useful. We must also consider that the sample was limited to a primary health care area, so there was a potential risk of selection bias, as the population of this area could differ from others. Therefore, these aspects must be considered when interpreting the external validity of our results. Another limitation is that, given the short baseline period and the type of study performed, causal conclusions on whether or not the observed increase in the use of psychopharmaceuticals is due to the pandemic or not cannot be drawn. However, our findings are statistically significant and may be relevant for actual clinical practice and future research.

Our research also had some strengths. One of the most important is that it was performed in a large primary health care area with a high population, so the sample is considerable and the validity of our findings, albeit limited, is also significant due to the size of the population analyzed. In this sense, another strength is that the data were extracted from an institutional database, so the risk of potential bias generated when using questionnaires was avoided. As the primary health care area was large, we could compare urban and rural areas, and areas with social transformation needs, with other areas. Finally, it is important to acknowledge that although some prescriptions were out of the reach of this research, the database contained information for 99.8% of the population, which increases the validity of our findings.

## 5. Conclusions

The consumption of the three subgroups of psychiatric drugs (anxiolytics, hypnotic-sedatives, and antidepressants) has increased since the beginning of the COVID-19 pandemic, especially in women and older people, two of the most vulnerable population groups. There is a risk of persistence in this situation. Thus, we should perform more research on the adequate use of these drugs during the pandemic. For future research, it would be interesting to investigate if the consumption of psychiatric drugs could be lowered using other nonpharmacology treatments. It could be interesting to perform this research using more extended periods or including trend analysis. We also think that it would be interesting to see if the use of psychiatric drugs was particularly high among residents of nursing homes. It could also be interesting to include the analysis of the antipsychotic drugs.

## Figures and Tables

**Table 1:** DHD of psychiatric drugs, regarding sex and age.

		DHD Anxiolytics			DHD Hypnotic-Sedatives			DHD Antidepressants		
Sex	Age Strata	COVID-19 Period	Pre-COVID-19 Period	Difference (%)	COVID-19 Period	Pre-COVID-19 Period	Difference (%)	COVID-19 Period	Pre-COVID-19 Period	Difference (%)
Women	25–34	17.8	14.2	25	3.1	2.7	15.	21.2	18.9	12
	35–44	42.6	36.9	15	9.6	8.6	11	44.8	45.0	−0.4
	45–54	74.3	70.8	5.1	26.9	24.6	9.2	99.7	97.4	2.4
	55–64	119.6	111.7	7.1	59.5	57.3	3.9	165.6	160.4	3.3
	65–74	153.3	143.0	7.2	113.1	108.3	4.4	229.1	220.2	4.1
	75–84	171.4	142.3	20	170.5	169.1	0.8	286.0	278.3	2.8
	>84	146.7	136.0	7.9	211.1	194.5	8.6	842.9	811.2	3.9
	Total	62.0	57.5	7.9	38.2	36.4	4.8	88.0	84.9	3.6
Men	25–34	11.9	11.6	2.8	2.8	1.9	46	8.9	8.7	2.2
	35–44	27.3	27.3	−0.1	5.0	4.8	5.2	18.2	17.9	1.3
	45–54	59.2	56.3	5.2	14.4	12.9	12	34.8	32.0	8.8
	55–64	64.6	58.1	11	31.5	29.0	8.5	59.4	57.2	3.9
	65–74	66.3	61.4	8.0	60.4	57.5	5.0	63.2	61.5	2.9
	75–84	74.3	72.5	2.4	96.9	92.7	4.6	101.5	99.3	2.2
	>84	82.7	74.6	11	120.1	113.2	6.0	112.3	108.9	3.1
	Total	32.7	30.8	6.	17.1	16.0	7.2	27.3	26.3	3.9

**Table 2:** Mean DHD of psychiatric drugs in persons under 65 and persons older than 65, in the pre-COVID-19 and the COVID-19 periods.

Drugs	Sex	Period	Age Strata	Mean DHD	S.D. *	*p*-Value **
Anxiolytics	Women	COVID	<65	63.6	43.9	0.01
			≥65	157.1	12.8	
		Pre-COVID	<65	58.4	42.4	0.02
			≥65	140.4	3.8	
	Men	COVID	<65	40.8	25.3	0.08
			≥65	74.4	8.2	
		Pre-COVID	<65	38.3	22.7	0.07
			≥65	69.5	7.1	
Hypnotic-sedatives	Women	COVID	<65	24.8	25.2	0.004
			≥65	164.9	49.2	
		Pre-COVID	<65	23.3	24.5	0.003
			≥65	157.23	44.3	
	Men	COVID	<65	13.4	13.0	0.005
			≥65	92.5	30.1	
		Pre-COVID	<65	12.1	12.1	0.004
			≥65	87.8	28.2	
Antidepressants	Women	COVID	<65	82.8	64.2	0.04
			≥65	452.6	339.2	
		Pre-COVID	<65	80.4	62.5	0.04
			≥65	436.5	325.7	
	Men	COVID	<65	30.3	22.2	0.019
			≥65	92.3	25.8	
		Pre-COVID	<65	28.9	21.1	0.017
			≥65	89.9	25.1	

* standard deviation ** Mann–Whitney test.

**Table 3:** Total consumption during the pre-COVID-19 and COVID-19 periods.

Consumption (DHD)	Period	Mean	S.D. *	*p*-Value **	Rosenthal Test
Anxiolytics	COVID	51.4	22.2	0.001	−0.78
	Pre-COVID	47.9	21.5		
Hypnotic-sedatives	COVID	29.4	16.2	0.001	−0.68
	Pre-COVID	27.9	15.6		
Antidepressants	COVID	61.4	39.1	0.003	−0.48
	Pre-COVID	60.1	38.6		

* standard deviation ** Wilcoxon test.

**Table 4:** Psychiatric drugs consumption in urban and rural areas during the pre-COVID-19 and COVID-19 periods.

	Rural Areas					Urban Areas			
Consumption (DHD)	Period	Mean	S.D. *	*p*-Value **	Rosenthal Test	Mean	S.D. *	*p*-Value **	Rosenthal Test
Anxiolytics	COVID	60.0	26.2	0.004	−0.84	47.2	19.0	0.001	−0.77
	Pre-COVID	56.2	26.3			43.9	17.8		
Hypnotic-sedatives	COVID	36.3	20.9	0.049	−0.57	25.9	12.2	0.001	−0.72
	Pre-COVID	34.7	20.4			24.5	11.6		
Antidepressants	COVID	78.2	47.9	0.131	−0.43	53.1	31.8	0.01	−0.52
	Pre-COVID	77.1	47.8			51.6	30.8		

* standard deviation ** Wilcoxon test.

**Table 5:** Psychiatric drugs consumption in areas with social transformation needs in the pre-COVID-19 and the COVID-19 period.

	Areas with Social Transformation Needs					Areas with No Social Transformation Needs			
Consumption (DHD)	Period	Mean	S.D. *	*p*-Value **	Rosenthal Test	Mean	S.D. *	*p*-Value **	Rosenthal Test
Anxiolytics	COVID	49.2	25.2	0.17	−0.55	51.89	21.9	0.001	−0.85
	Pre-COVID	46.9	23.4			48.18	21.5		
Hypnotic-sedatives	COVID	22.0	14.8	0.46	−0.29	30.84	16.2	0.001	−0.73
	Pre-COVID	21.5	13.8			29.21	15.8		
Antidepressants	COVID	36.7	32.6	0.45	−0.30	66.46	38.9	0.001	−0.61
	Pre-COVID	37.0	33.7			64.69	38.4		

* standard deviation ** Wilcoxon test.

**Table 6:** Psychiatric drugs consumption regarding sex in the pre-COVID-19 and the COVID-19 period.

	Women					Men			
Consumption (DHD)	Period	Mean	S.D. *	*p*-Value **	Rosenthal Test	Mean	S.D. *	*p*-Value **	Rosenthal Test
Anxiolytics	COVID	58.5	25.2	0.001	−0.83	44.4	16.5	0.002	−0.72
	Pre-COVID	54.6	24.6			41.4	15.8		
Hypnotic-sedatives	COVID	34.1	18.5	0.011	−0.60	24.6	12.2	0.001	−0.77
	Pre-COVID	32.8	17.4			23.1	12.1		
Antidepressants	COVID	78.5	41.4	0.064	−0.43	44.5	28.6	0.019	−0.55
	Pre-COVID	77.2	41.1			42.9	27.7		

* standard deviation ** Wilcoxon test.

**Table 7:** Psychiatric drugs consumption comparing men and women, urban and rural areas, and areas with social transformation needs and areas with no needs in the pre-COVID-19 and the COVID-19 period.

Consumption (DHD)	Period	Variable	Mean	S.D. *	*p*-Value **	Rosenthal Test
Anxiolytics	COVID	Women	58.5	25.2	0.04	−0.32
		Men	44.4	16.5		
	Pre-COVID	Women	54.6	24.6	0.06	−0.31
		Men	41.4	15.8		
Hypnotic-sedatives	COVID	Women	34.1	18.5	0.09	−0.28
		Men	24.6	12.2		
	Pre-COVID	Women	32.8	17.4	0.04	−0.33
		Men	23.1	12.1		
Antidepressants	COVID	Women	78.5	41.4	0.02	−0.37
		Men	44.5	28.6		
	Pre-COVID	Women	77.2	41.1	0.01	−0.42
		Men	42.9	27.7		
Anxiolytics	COVID	Rural	60.0	26.2	0.22	−0.20
		Urban	47.2	19.0		
	Pre-COVID	Rural	56.2	26.3	0.32	−0.16
		Urban	43.9	17.8		
Hypnotic-sedatives	COVID	Rural	36.3	20.9	0.14	−0.24
		Urban	25.9	12.2		
	Pre-COVID	Rural	34.7	20.4	0.14	−0.24
		Urban	24.5	11.6		
Antidepressants	COVID	Rural	78.2	47.9	0.05	−0.33
		Urban	53.1	31.8		
	Pre-COVID	Rural	77.1	47.8	0.05	−0.31
		Urban	51.6	30.8		
Anxiolytics	COVID	No STN	51.9	21.9	0.66	−0.07
		STN	49.2	25.2		
	Pre-COVID	No STN	48.2	21.5	0.98	−0.01
		STN	46.9	23.4		
Hypnotic-sedatives	COVID	No STN	30.8	16.2	0.10	−0.27
		STN	22.0	14.8		
	Pre-COVID	No STN	29.2	15.8	0.14	−0.24
		STN	21.5	13.8		
Antidepressants	COVID	No STN	66.5	38.9	0.02	−0.36
		STN	36.7	32.6		
	Pre-COVID	No STN	64.7	38.4	0.06	−0.31
		STN	37.0	33.7		

* standard deviation ** Mann–Whitney test; STN = area with social transformation needs.

## Data Availability

All data are contained within the article.
